# Adapted Virtual Reality Exergaming Using Off-the-Shelf Supplies for Poststroke Hemiparetic Arm Rehabilitation: Case Study

**DOI:** 10.2196/80721

**Published:** 2025-12-24

**Authors:** Sadie Hare, Jason Vice, Mary H Bowman, Ashley Wright, Raven Young, Mahmoud Ahmad, Maggie Logan, Byron Lai

**Affiliations:** 1 Department of Occupational Therapy University of Alabama at Birmingham Birmingham, AL United States; 2 Division of Pediatric Rehabilitation Medicine Department of Pediatrics University of Alabama at Birmingham Birmingham, AL United States

**Keywords:** active video game, augmented reality, disability, exercise, extended reality, physical activity, rehabilitation

## Abstract

**Background:**

Virtual reality (VR) can supplement exercise therapy for poststroke upper-arm hemiparesis, but treatments have been largely limited by specialized or costly equipment, hindering replicability and generalizability.

**Objective:**

This study examined the feasibility of using a commercially available bundle of VR supplies to improve hemiparetic arm function before and after an exergaming program in an individual post stroke.

**Methods:**

We conducted a pre-post case study (male, aged 72 years, chronic stroke) of a 20-day VR exergaming program (1-hour session per day) using a head-mounted display (Meta Quest 2), with adaptive software (WalkinVR) to boost and adjust in-game movements. Measures of upper-arm function were performed at preintervention (day 0), midintervention (day 10), and postintervention (day 21) and included the Wolf Motor Function Test (WMFT) and Disabilities of the Arm, Shoulder, and Hand Questionnaire (DASH). Data were descriptively analyzed.

**Results:**

The participant demonstrated improvement in the mean time to complete tasks of the WMFT by 70.5% (δ=11.73 s; preintervention mean time 16.63, SD 31 s; effect size=0.54) from preintervention to midintervention and 78% (δ=12.96 s; effect size=0.59) from preintervention to postintervention. WMFT mean functional ability score demonstrated an improvement of 18% (δ=0.46 points; preintervention mean score 2.67, SD 0.87 points; effect size=0.59) from preintervention to midintervention and 23% (δ=0.6 points; effect size=0.79) from preintervention to postintervention. Range of motion improved in all joints by an average of 35.64% (SD 20%) from preintervention to postintervention. DASH scores demonstrated minimal improvements across the intervention.

**Conclusions:**

VR exergaming with adaptive software could be an easy-to-adopt method for improving the functional ability of the hemiparetic arm among people post stroke. Improvements were potentially meaningful but warrant confirmation in more rigorous study designs.

## Introduction

A stroke results from occlusion of the blood supply to a part of the brain and often leads to many impairment-related functional limitations for those affected. Poststroke functional limitations hinder a person’s ability to perform their activities of daily living and other meaningful daily tasks. Specifically, damage in motor control areas or associated pathways can lead to decreased unilateral upper extremity function (ie, hemiparesis) [1,2]. Hemiparesis of an upper limb typically results in weakness of the affected limb, resulting in a limited range of motion and impaired ability to complete functional tasks. Upper-limb hemiparesis is a common impairment, making it a high-priority target for therapeutic interventions.

An effective method of regaining function in an affected limb is mirror therapy [3]. Mirror therapy can be described as involving the use of a mirror to hide the impaired limb of an individual while reflecting the unaffected one [4]. Asking the individual to move both limbs reflects the motion in the unaffected limb, providing visual feedback implying full movements in both arms. A specific mechanism for this feedback therapy is the activation of the mirror neuron system (MNS) [5-7]. The MNS is a theoretical compilation of neurons that are used when an individual performs a specific action. Most importantly, the system can be activated when simply observing a task being completed, particularly from a first-person view [8-10]. For example, mirror therapy is designed to facilitate neuroplasticity in motor areas affected by a stroke through visual presentation of actions that increase activity in the MNS [7,9]. However, since mirror therapy generally requires mass repetition of a simple task over a long period, individuals may struggle with maintaining adherence to the therapy prescription [5].

Game-based rehabilitation therapies are recognized for fostering high engagement among participants [11], and these interventions have been transformed by innovations in virtual reality (VR) technology [5]. VR is “a computer-generated environment in which people can feel and interact with various situations in three dimensions” [5]. There have been studies that have investigated the plausibility and effectiveness of combining MNS-based training with VR systems to create functional gains in upper extremity function among stroke survivors [5,9]. One study created a mirror-based VR rehabilitation system that provided unilateral and bilateral limb mirroring exercises in a fully immersive virtual environment [12]. After that intervention, the participants demonstrated significant improvements in their Fugl-Meyer Upper Extremity Assessment (FM-UE) scores. One of the measures on the FM-UE is proprioception, defined as the ability to perceive or be aware of the position and movement of the body [13]. In the case of VR mirror therapy for arm abduction, proprioception in the shoulder joint could be perceptually recalibrated in order to convince people post stroke that their motions are larger (or even smaller) than in reality [4]. Nevertheless, it is important to note that the precise mechanism (ie, MNS) underlying improvements following VR mirror therapy is still unclear. Meta-analysis has demonstrated that Mu suppression, an outcome often used to assess the effects of mirror therapy, was a valid indicator of the MNS [14]. However, recent investigations suggest the strength of Mu suppression as a mechanism could be confounded by other factors [15,16].

VR mirror therapy research for improving poststroke upper-arm hemiparesis appears promising but has been limited by complex setups, costly equipment, and a supplementation of nonspecified physical or occupational therapy treatment, hindering replicability and generalizability [17]. A recent meta-analysis found that VR mirror therapy was more effective than control groups [17]. For example, one study by Jo et al [18] demonstrated that a 360-degree immersive VR intervention was more effective than conventional mirror therapy versus a control group. However, the meta-analysis called for further investigations with larger randomized controlled trials, because the review included only 5 randomized controlled trials, totaling 148 people post stroke (a sample size of 30 people per study). To conduct large-scale trials, VR interventions will require easy-to-use methodologies that can facilitate adoption across a large variety of settings.

The goal of this study was to evaluate the feasibility of a low-cost, mirror neuron–based VR gaming protocol to improve upper extremity function in a person with chronic stroke. The protocol combined “off-the-shelf” supplies and software with an intervention dose that matched the average prescription given in mirror therapy. The VR headset included the Meta Quest 2, an all-in-one headset with built-in tracking, and a rhythmic movement-to-music game (Beat Saber), along with a computer and freely downloadable recalibration software (WalkinVR) to adapt conventional gaming for people with physical disabilities. The ease of obtaining study supplies (obtainable in any major electronics retailer) makes the intervention protocol capable of being easily implemented in various clinical and community settings.

Study purposes:

1. The primary purpose of this study was to examine the potential benefits of a short, intensive VR-adaptive therapeutic gaming intervention on hemiparetic stroke function and range of motion in an individual post stroke at midintervention and postintervention.
2. The second purpose of this study was to explore the feasibility of the study through intervention adherence, safety, and technical issues.
3. The third aim was to qualitatively describe the participant’s satisfaction with the program (acceptability of the program) through perceptions of intervention benefits, likes and dislikes, and recommendations for improvement.

## Methods

### Design and Overview

This case study was a pre-post trial design for a single participant. The intervention and data collection were conducted on-site at a university laboratory. The study lasted 4 weeks, which totaled 20 days of intervention. The study included both quantitative and qualitative procedures, analyzed separately (ie, not mixed methods). The study included quantitative outcomes that were measured before (day 0), in the middle (day 10), and after the 20-day intervention (total duration of 4 weeks, with intervention conducted only on weekdays). The study also included a qualitative postintervention interview. This case study followed the CARE (Case Report) guidelines while incorporating selected Single-Case Reporting Guideline in Behavioural Interventions (SCRIBE) elements relevant to behavioral intervention research (eg, description of context, participant flow, and intervention fidelity). This hybrid approach was used to maximize transparency and ethics (no multiple baseline or follow-up to minimize participant burden and no withdrawal phase to test a washout effect on outcomes).

### Participants

The participant was chosen based on the following inclusion criteria: (1) aged ≥18 years, (2) chronic stroke (time since onset ≥6 months), (3) hemiparesis with a minimal degree of functional ability of the affected arm (manual muscle test score ≥2), (4) access to transportation to the laboratory, and (5) ability to communicate in English. Exclusion criteria included (1) having a health condition that prevented participation in moderate-intensity exercise and (2) susceptibility to VR-induced motion sickness. The participant was recruited via word of mouth through a rehabilitation research collaborative at the university. The rationale for including only a single participant was due to time and effort constraints, since the project needed to be completed within a university semester.

### Equipment and Setup

**Figure 1:**
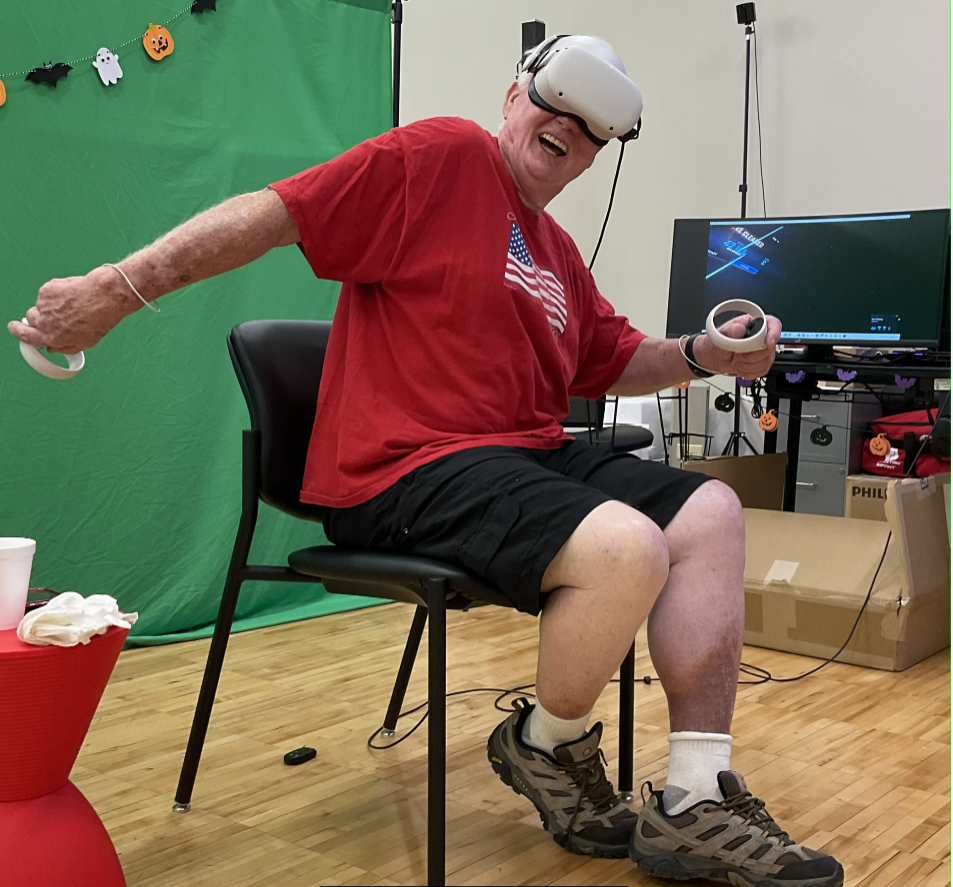
Demonstration of the participant post stroke playing Beat Saber.

Study equipment included a Meta Quest 2 that was connected to a desktop gaming computer through a high-speed USB cable (Meta Link Cable). The gaming computer included VR recalibration software, WalkinVR, through the Steam gaming platform. WalkinVR is a free-to-download application on Steam that allows VR gaming to be fully adapted for a person with a disability. When used for research, WalkinVR requires a fee for use and technical support. WalkinVR features include, but are not limited to, physical boosts or projections of small movements in real life to big movements in the VR environment and repositioning of the headset or handheld controllers in the digital space. Additional software included the VR game Beat Saber, which was installed on the desktop computer. The participant wore the Quest 2 while seated in an armless chair (shown in Figure 1).

### Intervention

The intervention prescription consisted of active gaming for 60 minutes per day, 4 times a week, for a total of 5 weeks. The intensity (ie, difficulty) of the gameplay ranged from “easy” to “hard.” The intensity and the level were selected by the participant. At each intervention visit, 2 research staff members helped the participant equip the VR headset, set up the gaming system, and supervise the exercise session. Research staff also provided occasional verbal praise for successful task completion and encouraged the participant to minimize downtime between completing one level and selecting another to play. On the first day of exercise, research staff calibrated the WalkinVR software for the needs of the participant. Calibration included 3 steps. First, the participant was instructed to maintain a neutral arm position, with elbow joints held to the side of their body, in a comfortable resting position, with the forearm pointed forward as straight as possible. Second, research staff used the WalkinVR software on the computer to adjust the controller position of the affected arm to match the position of the unaffected arm in the digital VR environment. Third, the participant was asked to play an easy level in Beat Saber. During calibration, if the participant had insufficient reach in the game to successfully complete the box-chopping tasks, real-time adjustments of controller position were made in WalkinVR until successful gameplay was achieved. As a means of intervention progression, positional assistance in the affected arm from WalkinVR was gradually reduced over time. Research staff were instructed to make minor reductions in positional assistance every few days, without making box-chopping too difficult (ensuring that the participant is chopping blocks with at least a 70% success rate). To avoid boredom from repeating the same levels that were provided in the base version of the game, the participant was allowed to request that the staff purchase additional levels in Beat Saber (song packs).

### Measures and Analyses

#### Quantitative

Quantitative data were collected on-site at preintervention (day 0), midintervention (day 10), and postintervention (after day 20). Primary aim outcomes included (1) the Wolf Motor Function Test (WMFT) and (2) the Disabilities of the Arm, Shoulder, and Hand Questionnaire (DASH). Secondary outcomes were the upper extremity range of motion of the hemiparetic side as measured by goniometry. The WMFT includes 15 tasks, and participants are instructed to complete tasks as quickly as possible. The first 6 tasks of the WMFT include timed joint-segment movements, whereas tasks 7 to 15 include integrative functional movements. For this study, task 7 (the maximum weight a person can lift onto the table) and task 14 (grip strength) were excluded because musculoskeletal strength measures were not relevant to the study goals. The WMFT provides a total score based on average task completion time, capped at 120 seconds per task. It can also be rated on a functional ability scale from 0 to 5—“0” means the participant “does not attempt with the involved arm,” and “5” means the “arm does participate and the movement appears to be normal.” Primary and secondary outcome measures were conducted by an occupational therapy graduate student, who was instructed by a senior occupational therapist. Functional ability scores were rated by a senior therapist not involved in the intervention.

Because this study involved a single participant, quantitative data were analyzed descriptively. Means, SDs, percent change, and standardized difference scores (effect size) were calculated to describe the magnitude of change across time points. The standardized difference was computed as the mean change divided by the pooled SD between 2 time points. This approach was used only to provide a descriptive indication of change, rather than for statistical inference. Minimal clinically important difference (MCID) and minimal detectable change (MDC) values from prior validation studies were reported alongside the descriptive metrics to support interpretation of the clinical relevance of observed changes.

Tertiary feasibility outcomes included attendance, technology issues, and unanticipated problems. Attendance was defined as the number of sessions attended and completed divided by the number of prescribed sessions. An arbitrary value of 70% attendance was considered “acceptable.” Technology issues that were encountered by the participant during each session were documented by research staff. Problems and adverse events were recorded and reported in accordance with university procedures.

#### Qualitative

After completion of the intervention, the participant underwent a semistructured interview (in-person) with the lead interventionist (SH) at the end of the program. The qualitative component of this study was an intrinsic case study design [19,20] to explore the lived experience of a single participant who completed the program. The specific philosophical assumptions that underpinned the qualitative study methods were critical realism, from the ontological perspective, and interpretivism, from the epistemological perspective [21]. These assumptions allowed the researchers to recognize that the participant will perceive a reality when reporting responses, but this reality could be subtly enhanced by the interaction between participant and interviewer and the act of interpreting the data. Underpinned by an interpretivist epistemology, our aim was to understand how the participant makes meaning of exercising in VR post stroke, rather than to quantify frequency of codes or seek a single “true” account. The interview included 10 overarching questions with several follow-up questions. The questions probed participants’ overall views of the program, likes and dislikes, experiences using the VR equipment for upper extremity exercise, comparison with previous therapy experience, barriers and facilitators to meeting the intervention prescription, preferences, and recommendations to enhance the protocol for a future trial. The interviewer was trained by a qualitative expert in disability and exercise (BL), who had conducted more than 400 interviews related to exercise and disability. The interview was audio recorded and transcribed for analysis by a member of the research team.

Qualitative analysis was framed by Braun and Clarke’s [22,23] 6-step latent thematic analysis approach. First, the analyst familiarized himself with the data by reading the transcript multiple times and noting early impressions and reflexive insights about tone, emotion, and context. Second, initial codes were generated using artificial intelligence (ChatGPT) on a segment-by-segment basis to identify relevant units of meaning to describe actions, perceptions, or feelings. Third, codes were aggregated and clustered using ChatGPT to form one level of overarching categories (ie, themes). The rationale for including only one level of themes was due to only having one interview; an attempt to provide fewer, more concentrated themes [24] rather than abundant but watered-down themes. Fourth, the qualitative expert analyst cross-checked and revised the resultant codes and themes with the case transcription for accuracy (ie, resultant codes similar to what the analyst would have generated) and relevancy (ie, ensuring that codes and themes aligned with philosophical assumptions and the study objective). Fifth, themes were revised again to more clearly define the central concept of the supporting codes, and additional participant quotes were sought to strengthen concepts. Quotations and codes were selected for their richness and relevance, rather than frequency. Sixth, the results were presented in an interpretive commentary with participant quotations to support the concept. To enhance rigor throughout this process, reflexivity was maintained through memoing and keeping an audit trail for iterations of themes and codes. The transcription was performed by ChatGPT and double-checked for accuracy by an author.

### Ethical Considerations

This study was conducted in accordance with case study guidelines set by the Institutional Review Board for Human Use at the University of Alabama at Birmingham, and approval for the study was granted (IRB-300010445). Written informed consent was obtained from the participant prior to participation. Identifying information (eg, name, initials, hospital numbers) of the case participant was omitted from publication, and data were deidentified and stored using an alpha numeric code.The participant was compensated at the end of the study with a US $50 gift card.

## Results

The participant, referred to as “Chop,” was a 72-year-old White male, 5’11” tall and weighing 220 lb. He was 3 years post stroke and used a cane as a primary mobility device and a power wheelchair during prolonged daily activities. He had hemiparesis affecting his left, nondominant side. He had no cognitive, visual, or hearing impairment preventing him from completing the intervention.

### Feasibility Metrics (Adherence, Safety and Technical Issues)

Chop attended and completed 20 intervention sessions (20/20, 100% attendance), with only one additional song pack purchase in Beat Saber. The participant rescheduled 5 sessions (5/20, 25%). No adverse events or problems were experienced by the participant during the intervention. As for technical issues, the participant reported the following equipment-related problems: (1) the VR headset had a slight weight, exerting mild pressure on the participant’s face, despite using a head-strap accessory, and (2) the controller was challenging to grasp during exercises for the nondominant (affected) hand, even with the hand-knuckle strap accessory.

### Wolf Motor Function Test

**Figure 2:**
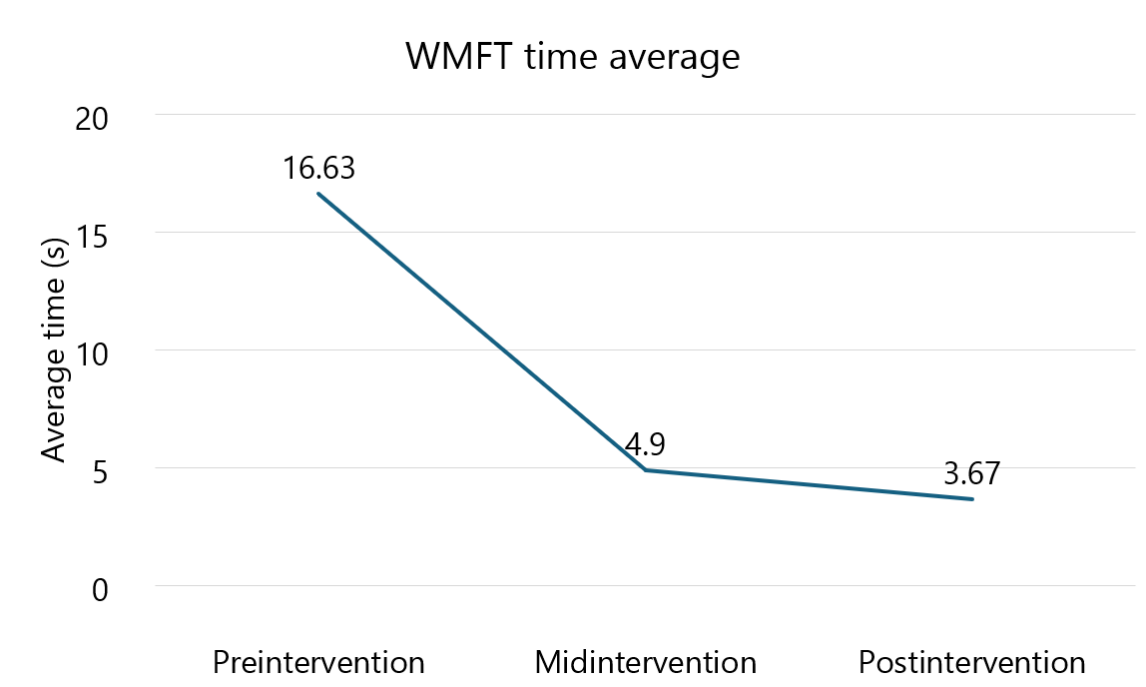
Average time to complete tasks for the Wolf Motor Function Test (WMFT).

**Figure 3:**
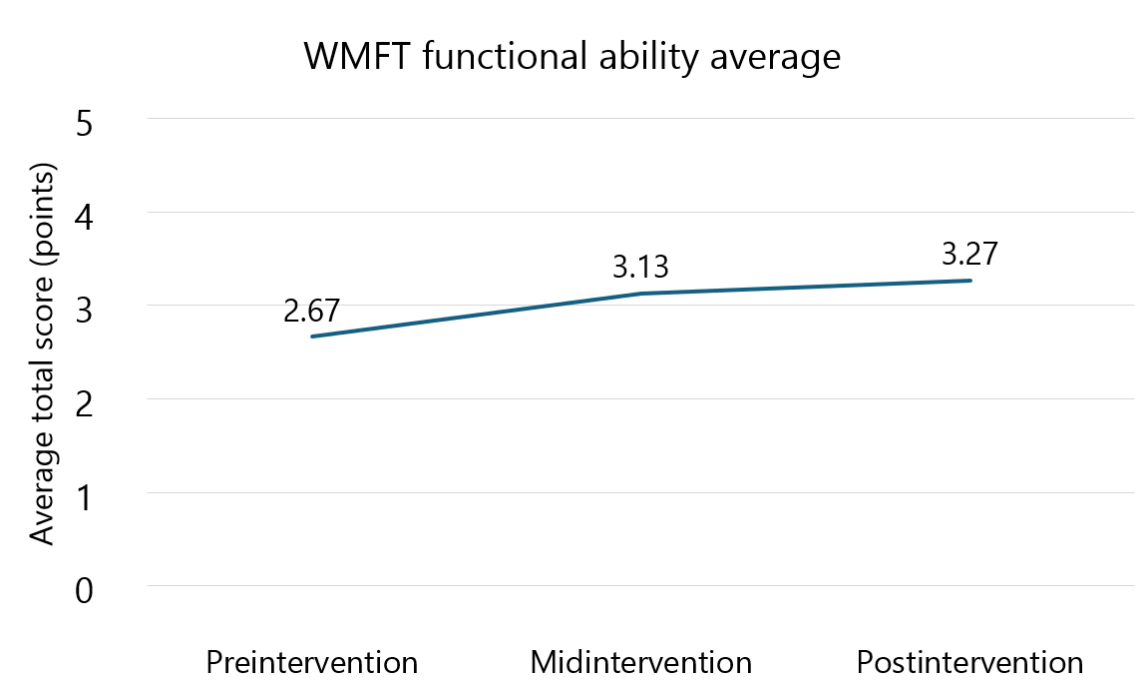
Average Wolf Motor Function Test (WMFT) functional ability score.

Figures 2 and 3 display the results for the average time to complete tasks and the functional ability score for the WMFT, respectively. The results for each task of the WMFT are shown in Table 1 (time to complete) and Table 2 (task score). Results demonstrated that the average time to complete tasks improved by 70.5% (δ=11.73 s; preintervention mean time 16.63, SD 31 s; effect size=0.54) from preintervention to midintervention and 78% (δ=12.96 s; effect size=0.59) from preintervention to postintervention. A 25% (δ=1.23 s; midintervention mean time 4.9, SD 4 s; effect size=0.33) improvement was observed from midintervention to postintervention. All of these changes exceeded an MDC_95%_ of 0.7 seconds [25], as well as an MCID of small effect. Results for functional ability score demonstrated improvements of 18% (δ=0.46 points; preintervention mean score 2.67, SD 0.82 points; effect size=0.59) from preintervention to midintervention and 23% (δ=0.6 points; effect size=0.79) from preintervention to postintervention. A 4% (δ=0.14 points; midintervention mean score 3.13, SD 0.74 points; effect size=0.19) improvement was observed from midintervention to postintervention. All of these changes surpassed an MDC_95%_ of 0.1 points [25]. Changes from preintervention to midintervention and preintervention to postintervention exceeded an MCID of small effect, but the change from midintervention to postintervention did not.

**Table 1:** Wolf Motor Function Test time.

Item	Preintervention (seconds)	Midpoint (seconds)	Postintervention (seconds)
1	2.75	1.25	1.11
2	17.56	4.78	1.34
3	120	12.31	14.03
4	43.16	9.2	2.88
5	1.38	0.84	0.79
6	2.34	1.82	1.37
7	N/A^a^	N/A	N/A
8	1.09	1.03	0.85
9	2.78	2.68	2.09
10	2.91	1.71	1.88
11	4.19	2.03	1.87
12	20.34	5.87	4.87
13	9	7.88	6.25
14	N/A	N/A	N/A
15	6	6.25	5.16
16	10.79	11.6	8.22
17	5.1	4.25	2.38
Mean (SD)	16.63 (30.7)	4.9 (3.9)	3.67 (3.6)

^a^N/A: not applicable.

**Table 2:** Wolf Motor Function Test score.

Item	Preintervention	Midpoint	Postintervention
1	3	3	3
2	2	3	3
3	1	2	2
4	2	2	3
5	3	4	4
6	3	4	4
7	N/A^a^	N/A	N/A
8	2	3	3
9	4	3	4
10	3	4	4
11	3	4	4
12	2	4	4
13	3	3	2
14	N/A	N/A	N/A
15	4	3	3
16	3	2	3
17	2	3	3
Mean (SD)	2.67 (0.82)	3.13 (0.74)	3.27 (0.70)

^a^N/A: not applicable.

### Disabilities of the Arm, Shoulder, and Hand Questionnaire

Results demonstrated that the total DASH score, from preintervention (41.67 points) to midintervention (36.68 points), improved by 11.98% (δ=4.99 points; effect size=0.23) and 10% (δ=4.17 points; effect size=0.18) from preintervention to postintervention (37.5 points). From midintervention to postintervention, a change of 2.25% (δ=0.83 points; effect size=0.03) was observed. None of these changes surpassed an MDC_95%_ of 12.75 total score points [26].

### Range of Motion

The results for Chop’s affected arms’ range of motion preintervention and postintervention can be found in Table 3. Results demonstrated that range of motion improved in all joints by an average of 35.67% (SD 20%). Favorable changes in range of motion (ie, improvements toward the normal range of motion for a given joint) were observed in all joints. Notable changes were observed for shoulder flexion (67%), elbow flexion (50%), hand supination (40%), and elbow extension (35%). Minimal improvements were observed for arm abduction (9%). Hand pronation improved minimally (10%), but a normal range of 90 degrees was achieved at postintervention.

**Table 3:** Range of motion for hemiparetic arm joints.

	Preintervention (degree)	Postintervention (degree)	Change, degree (%)
Left shoulder flexion	55	92	37 (67)
Left elbow extension	40	26	–14 (–35)^a^
Left elbow flexion	80	120	40 (50)
Left arm abduction	110	120	10 (9)
Left hand pronation	80	90	10 (13)
Left hand supination	50	70	20 (40)

^a^Negative values indicate a reduction in joint range of motion from preintervention to postintervention.

### Qualitative Results

#### Case Description

Chop was a 72-year-old White male living in a suburban area in Alabama, United States. Chop used a standard cane as a primary means of mobility and used a power wheelchair when traveling in new or high-activity areas. Chop did not participate in any exercise before this study began. He completed inpatient occupational and physical therapy immediately following his stroke and outpatient therapy periodically during the first year post stroke. In the year before this study, Chop did not participate in formal therapy, feeling he had reached a plateau in functional ability. As a grandparent, he spent much of his time with and entertained his grandchildren. He saw himself as a handyman, enjoying fixing things, and spent his free time working on his car and fixing appliances. After reaching a functional plateau, Chop relied on compensatory strategies and adaptive equipment for daily tasks like dressing, bathing, toileting, socializing, and driving. Qualitative analysis resulted in 4 themes that are displayed in Table 4 and described below.

**Table 4:** Qualitative results.

Theme	Codes	Supporting quotes
Curiosity into confidence: “Not sure what it was, but it was exciting”	Initial curiosity creates feelings of excitement Confidence is questionable prior to participation Early physical exertion leads to thoughts of withdrawal Persistence was supported by enjoyment with the program and his commitment to the study team Confidence bolstered by 2 weeks of successful repetition	“I really wasn’t sure. I think I didn’t really know for sure what it was... I mean, it was exciting.” “I think I was confident I could do it. I just wasn’t sure... Like anything, I think I can do them all. Everything I think I can do, but I can’t always do it.” “It did surprise me a little. It surprised me that I was doing that. Really, you know, somebody don’t really play them kind of games and stuff, you know? That was surprising, but yeah, I think it’s pretty good.” Interviewer: Was it difficult to do 60 minutes of it four days a week? “No, it wasn’t difficult, you know. It just, no, not really. I mean, I guess I did get a little tired.” “[first week of the program] I said, I ain’t going back to see these girls I’ve had enough of. I got three more weeks left? No, I mean, I just thought, this is wild. This is wild to me, you know... I ain’t played a lot of these games, you know. But it was fun.”
Redefining rehabilitation through play: “It wasn’t boring like therapy”	Gamification transforms past perceptions of therapy Virtual reality is innovative for its higher cognitive demand and difficulty	“I guess it [conventional therapy] worked, but you know, it’s always boring. Yeah. You know, and I mean, comparing the two [conventional versus virtual reality], it’s just like night and day... I think it causes you to have more mental reactions to help you move.” “Well, I mean, I liked it all... I mean, I just enjoyed it. I didn’t mind going over and doing it. I mean, it was a lot of fun.”
Reclaiming independence: “I could tie my boots again”	Improved function and range of motion of the affected arm Functional improvements fostered benefits to daily activities Newly found modifications for daily activities Early improvements	“I was putting on my socks a lot easier than I was.” “I put on some boots... Well, half boots. Laced them and actually tied them... It’s been a while since I put on anything. Most of my stuff is all slip-on stuff now.” “After that first week, I might have tested a little more movement. I would notice things. I’m doing this [demonstrates movement], where it normally would have been hard to do.” “One thing I did get out of... Getting out of the bed, when I’d get over on my side, I would push myself up more with this arm than I have been doing. “Seems like I have been doing better with this arm [affected arm] on things I’m working on, where normally it’s just running all over the place. You know, be still while you do this.”

#### Theme 1: Curiosity Into Confidence

“Not sure what it was, but it was exciting.” Chop began the program with initial curiosity and uncertainty, which gradually progressed to growing confidence and engagement. Before starting the program, he perceived VR to be mysterious and exciting, a novelty period, which drove him to pursue the program despite internal struggles. However, initial excitement was coupled with internal uncertainty regarding his confidence to successfully engage in the program using his affected arm. Uncertainty was also affected by a lack of experience playing video games, and this uncertainty lingered through the first week of the program. Moreover, the first week of the program brought additional internal tension between the desire to persist and physical fatigue from the exercise. Chop described early physical exertion, particularly related to his affected arm, which led to thoughts of withdrawing from the program: “I said, I ain’t going back to see these girls. I’ve had enough of it.” These thoughts were outweighed by the enjoyment of the program, his perceived commitment to the study team, and growing confidence after 2 weeks of successful completion of his exercises. As repetition accumulated over the first 2 weeks, the participant reported improved confidence and described surprise at his own competence and adaptation to the virtual environment: “It did surprise me a little. It surprised me that I was doing that. Really, you know, somebody don’t really play them kind of games and stuff, you know?”

#### Theme 2: Redefining Rehabilitation Through Play

“It wasn’t boring like therapy.” This theme reveals how Chop’s understanding of therapy shifted from monotonous, clinical exercise to stimulating and playful engagement. He framed conventional therapy as “boring” and repetitive, but perceived VR as “like night and day.” The gamified environment demanded higher cognitive processing—reacting to visual and auditory cues, anticipating movement, and achieving performance feedback. This experience transformed his perception of rehabilitation from a medical obligation into a mentally engaging activity. When asked about conventional therapy, he explained:

“Well, I guess it [conventional therapy] worked but, you know, it was always boring. If I compare the therapy I’ve had to this VR kind, it’s like night and day. I think it [VR] causes you to have more of a mental reaction to help you move, move of a natural reaction rather than a repetitive and conscious effort to try to do something difficult.”

#### Theme 3: Reclaiming Independence

“I could tie my boots again.” Chop reported several improvements in the functional ability of his affected arm, which he noticed while performing daily tasks. Improvements included range of motion and control of his affected arm, which would previously be “running all over the place.” He reported accomplishing tasks he hadn’t been able to do since the stroke onset, such as independently tying his shoes with laces, easier bed mobility, working on his car, and dressing. After being able to tie his laced boots for the first time in years, the participant told his wife, “Look here, I put these on and tied them!” Additionally, he discovered new and efficient modifications to daily activities: “I would push myself up more with this [affected] arm than I have been doing.” Chop reported that he could do more at home and that those benefits were observed as early as the second week of the intervention.

#### Theme 4: Motivation Through Flow and Mastery

“It was a challenge, and that was good.” Chop repeatedly described VR exercise as an enjoyable challenge that kept him engaged. Successful gameplay demanded constant visual and cognitive attention to quickly react to in-game tasks, creating a mental state of performance without thought (referred to as “flow”): “It was happening so fast you didn’t have time to think about it, you know? You’d just done it.” Chop’s full attention was placed on gameplay performance, and this was supported by visual immersion in a virtual world and a high level of enjoyment in gameplay. These components distracted Chop from real-world movements of his affected arm, which allowed him to push past his limits:

“I feel like it was more helpful that I couldn’t see my arm in real life because it makes you feel like you’re moving your arm all the way. I think it’s because I could do a little bit more with it based on what I was seeing on the screen. It sort of tricks your brain.”

Chop reported that gameplay was difficult due to the physical fatigue of his affected arm. His main challenge was achieving complete accuracy in hitting all in-game boxes. His primary goal was to enhance his accuracy to hit blocks and maintain a high streak over time (repeated successful hits on blocks without failing). Chop explained why maintaining accuracy was the biggest challenge: “getting my arm up high enough and keep from getting so tired after playing for a while and after moving for so long. I never really move my left arm that much, especially not all in one hour.” Instead of deterring participation, gameplay difficulty was met by Chop’s firm resolve to persevere and master the challenges. Perseverance was supported by functional improvements he observed throughout the program, where Chop would engage in motivational self-talk: “I felt like even after the end of two weeks I was getting more function in my left arm. Each week I would tell myself that I could do better than the week before.”

## Discussion

### Overview and Principal Results

This study investigated the preliminary effect of a 20-day VR program to improve the affected upper limb function of a person post stroke with hemiparesis. The novel aspect of this study was the inclusion of adaptive VR software (free to download) to simulate a mirror therapy intervention, as well as the inclusion of low-cost technology that can be purchased at any major electronics retailer.

Quantitative findings demonstrated moderate, clinically meaningful improvements in the functional ability of the participant as measured by the WMFT, as well as a small, likely nonmeaningful change in self-reported functional ability as measured by the DASH. For the WMFT, a remarkable 70.5% (δ=11.73 s; preintervention mean time 16.63, SD 31 s) improvement in task-affected arm performance was observed at the midpoint of the intervention, approximately 10 days of intervention. Without a control group and a larger sample, it is difficult to compare the absolute improvement change to previous investigations. Nevertheless, moderate effects indicated by the WMFT were comparable to the moderate effects observed from conventional mirror therapy interventions [3]. Similar interpretations can be made for the WMFT functional ability findings [27-29]. WMFT improvements for this study appeared lower than the strong effect (effect size=0.84) on upper extremity function, measured by the FM-UE, which was reported in a meta-analysis of 4 other VR mirror therapy interventions [17]. These differences could potentially be explained by a few factors. First, past studies often supplemented VR intervention with physical or occupational therapy. Second, past VR interventions often conducted task-specific practice using specialized games that targeted upper extremity function, as opposed to using an “off-the-shelf” game that was not designed for therapeutic purposes. Third, differences could be due to the different measures of upper extremity function that were used (WMFT vs FM-UE). Fourth, previous interventions used different VR systems (eg, addition of specialized controllers to a head-mounted display or interactive television applications). Fifth, the participant in this study was not in the acute stage of recovery (<2 years post stroke), which could potentially dampen gains in physical function.

Of note, this study found improvements in range of motion, which indicated that WMFT improvements stemmed from the shoulder, elbow, and forearm joints. Interestingly, improvements in performance task time and functional ability score seemed much smaller from midintervention to postintervention. Collectively, these findings indicate that the intervention has a potentially moderate strength effect on affected arm functional ability at an even shorter intervention duration than originally planned. A moderate effect can likely be experienced following a brief 10-day intervention. A shorter intervention duration has obvious benefits, including reduced cost and burden, but this warrants investigation in a randomized controlled efficacy trial.

The qualitative findings expanded upon quantitative findings and, most importantly, identified potential mechanisms that led to the success of the intervention. The participant reported a physiological burden from the intervention, which was overcome by a few factors. The participant reported greater engagement in the exercise movements of the intervention compared to what he had previously experienced in conventional therapy. This was due to immersion within a digital environment, the enjoyment of the game box–chopping activity, the accompanied music, and the performance feedback reward system that put him in a state of “flow.” Moreover, the participant reported noticeable gains in the function of the affected arm, which led to favorable changes in his daily activities.

While these findings are encouraging, a notable limitation of VR mirror-simulated exergaming is that it requires moderate function of the affected arm to complete the in-game tasks successfully. Whereas conventional mirror therapy is likely beneficial when impairment of the affected arm is severe, and simply observing the nonaffected arm can stimulate small movements in the affected arm. Moreover, the study findings had numerous limitations, which warrant further investigation in a randomized controlled trial.

### Limitations

This proof-of-concept case study had limitations. First, the study included only a single case, which impedes the findings from any form of representativeness or generalizability. Second, the outcome assessor was not blinded, which could have affected the results. Third, the study did not have a control group. Including a control group or other kinds of control would help determine whether the observed improvements were due to the intervention itself rather than natural recovery, motivation, or other external factors. Fourth, this study did not include a repeated baseline design to allow stabilization of baseline outcomes prior to testing effects. Fifth, this study did not include a washout phase to test either outcome sustainability or a repeated intervention effect. Therefore, study findings should be interpreted with caution.

### Conclusion

This study identified a low-resource protocol for VR exergaming with adaptive software to improve the hemiparetic arm function of people post stroke. The identification of an easy-to-adopt protocol could help address a key limitation in VR mirror therapy research: small sample sizes and nonconfirmatory randomized clinical trials. Although favorable findings were observed in this case study, these findings need to be further explored in a pilot randomized controlled trial before larger investigations into efficacy or effectiveness are warranted.

## Data Availability

All data generated or analyzed during this study are included in this published paper.

## Abbreviations

CARE: Case Report
DASH: Disabilities of the Arm, Shoulder, and Hand Questionnaire
FM-UE: Fugl-Meyer Upper Extremity Assessment
MCID: minimal clinically important difference
MDC: minimal detectable change
MNS: mirror neuron system
SCRIBE: Single-Case Reporting Guideline in Behavioural Interventions
VR: virtual reality
WMFT: Wolf Motor Function Test
